# Recent advances in the study of NADC34-like porcine reproductive and respiratory syndrome virus in China

**DOI:** 10.3389/fmicb.2022.950402

**Published:** 2022-07-22

**Authors:** Hong-zhe Zhao, Feng-xue Wang, Xiao-yu Han, Hao Guo, Chun-yu Liu, Li-na Hou, Ya-xin Wang, Hui Zheng, Lu Wang, Yong-jun Wen

**Affiliations:** Key Laboratory for Clinical Diagnosis and Treatment of Animal Diseases of Ministry of Agriculture, College of Veterinary Medicine, Inner Mongolia Agricultural University, Hohhot, China

**Keywords:** PRRSV, NADC34-like, pathogenicity, RFLP, recombination

## Abstract

Since porcine reproductive and respiratory syndrome virus (PRRSV) was first described in China in 1996, several genetically distinct strains of PRRSV have emerged with varying pathogenicity and severity, thereby making the prevention and control of PRRS more difficult in China and worldwide. Between 2017 and 2021, the detection rate of NADC34-like strain in China increased. To date, NADC34-like strains have spread to 10 Chinese provinces and have thus developed different degrees of pathogenicity and mortality. In this review, we summarize the history of NADC34-like strains in China and clarify the prevalence, genomic characteristics, restriction fragment length polymorphisms, recombination, pathogenicity, and vaccine status of this strain in China. In so doing, this study aims to provide a basis for the further development of prevention and control measures targeting the NADC34-like strain.

## Introduction

Porcine reproductive and respiratory syndrome virus (PRRSV) is the causative agent of sow abortion, boar sperm deformity, and piglet respiratory disease (Collins et al., 1992; Meulenberg et al., 1993). In the United States, PRRSV outbreaks lead to annual economic losses of US$ 664 million, which is a huge blow to the swine industry in various countries (Dwivedi et al., 2012). There are two PRRSV genotypes: PRRSV-1 is represented by strain Lelystad, which was discovered in the Netherlands in 1991 (Wensvoort et al., 1991), while PRRSV-2 is represented by strain VR2332, which was discovered in the United States in 1992 (Collins et al., 1992). The homology between the two representative strains is approximately 60% (Nelsen et al., 1999; Kuhn et al., 2016). However, since the identification and characterization of prototype PRRSVs for PRRSV-1 and PRRSV-2, new variants of PRRSVs have evolved, and PRRSV outbreaks with increasingly divergent and virulent phenotypes have been reported (Kappes and Faaberg, 2015).

Before 2006, the main epidemic strain in China was classic PRRSV, represented by strain CH-1a, which was first isolated in 1996 (GenBank: AY032626). In 2006, an outbreak of PRRSV (HP-PRRSV) characterized by high mortality, high fever, and high abortion rates devastated the Chinese swine industry (Tian et al., 2007). Between 2006 and 2014, HP-PRRSV was the main epidemic strain of PRRSV in China (Gao et al., 2017). Since 2014, the proportion of NADC30-like strains detected in China has increased (Xie S. et al., 2020; Liu et al., 2021), and NADC30-like strains are currently some of the main epidemic strains in China (Ma et al., 2022). However, in 2018, the NADC34-like strain was discovered in Liaoning, China, and gradually became the dominant strain (Zhang et al., 2018; Xu et al., 2022). Genetic analyses show that the population genetic diversity of NADC30-like PRRSV is declining year on year, while the genetic diversity of NADC34-like PRRSV is increasing (Yu et al., 2020). To date, NADC34-like strain has been reported in 10 Chinese provinces and is threatening to become an epidemic in China. To provide a basis for improved PRRS prevention and control, we summarized and analyzed the genomes of NADC34-like strains in GenBank and reviewed the prevalence, pathogenicity, and clinical symptoms of NADC34-like strains, as well as their genomic and recombination characteristics.

## Prevalence of NADC34-like strains hints at dominance in China

**FIGURE 1:**
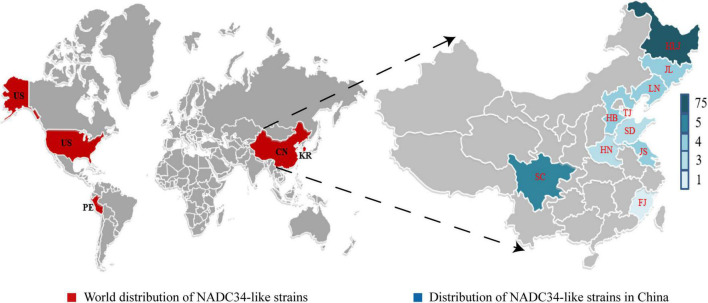
Distribution of NADC34-like strains worldwide and in China. Maps show the four countries and 10 Chinese provinces where NADC34-like strains have been identified to date: China, the United States, Peru, and South Korea. On the world map, countries known to harbor NADC34-like strains are colored red. On the map of China, provinces known to harbor NADC34-like strains are colored blue, with color intensity increasing with the number of unique NADC34-like strains identified. HLJ, Heilongjiang Province; JL, Jilin Province; LN, Liaoning Province; TJ, Tianjin City; HB, Hebei Province; SD, Shandong Province; HN, Henan Province; JS, Jiangsu Province; FJ, Fujian Province; SC, Sichuan Province. Maps were obtained from yourfreetemplates.com.

The PRRSV 1-7-4 (NADC34) strain was first reported in the United States in 2014 and was named IA/2014/NADC34 (GenBank: MG860516) (van Geelen et al., 2018). Various PRRSV 1-7-4 (NADC34-like) strains were also identified in 2015–2017 in Peru (Ramirez et al., 2019). Recently, the whole genome of an NADC34-like strain was reported from South Korea, implying that this strain was already prevalent in South Korea in 2017; the South Korean NADC34-like strain was obtained *via* the recombination of NADC34 (major parent) and NADC30 (minor parent) (Kim et al., 2022). Also in 2017, two NADC34-like strains (named LNWK96 and LNWK130) were isolated in Liaoning, China, and whole-genome sequencing indicated that these were chimeric viruses (Zhang et al., 2018). Between 2017 and 2021, NADC34-like strains spread to 10 Chinese provinces, with the highest rates of identification in Heilongjiang Province (Bao and Li, 2021; Sun et al., 2022; Xu et al., 2022; Zhao et al., 2022). Importantly, the lack of reports of NADC34-like strains in other Chinese provinces may be due to insufficient sampling rather than the absence of the virus. Despite this uncertainty, it is clear that NADC34-like strains are more widespread in northern China than in southern China (Figure 1). Between the period 2017–2021, detection rates of NADC34-like strains soared from 3% in 2017 to 11.5% in 2020 and an astonishing 28.6% in 2021 (Xu et al., 2022). At the same time, in 2021, the distribution of PRRSV strains at a large pig farm in China was 64.19% NADC30-like, 20.24% NADC34-like, 13.10% HP-like, 1.19% CH-1a-like PRRSV, and 1.19% QYYZ-like (Li et al., 2021). These previous studies show that NADC34-like strain is now the main epidemic strain in some areas of China, and this strain appears to be spreading across the country. It is possible that NADC34-like strains will become the main epidemic strain of PRRSV in China in the future. In 2021, the PRRSV strain RFLP 1-4-4 Lineage 1c Variant (NADC34-like), which caused high mortality in piglets and finishing pigs, was reported in the United States; this strain is affecting most pig farms in the Midwestern United States (Kikuti et al., 2021). The emergence of similar highly pathogenic strains has not yet been reported in China.

## NADC34-like strains have a unique evolutionary clade and molecular marker

**FIGURE 2:**
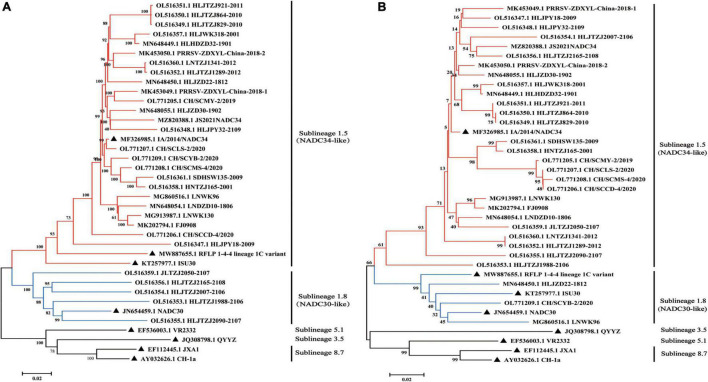
Phylogenetic tree based on the whole-genome and ORF5-gene sequences of NADC34-like strains from China. **(A)** Phylogenetic analysis of NADC34-like strains in China and reference viruses based on whole-genome sequences. **(B)** Phylogenetic analysis of NADC34-like strains in China and reference viruses based on ORF5-gene sequences. Most of the NADC34-like strains isolated in China belonged to Sublineage 1.5, but a few recombinant strains fell into Sublineage 1.8. The phylogenetic tree was constructed using the distance-based neighbor-joining algorithm in Mega7 (Kumar et al., 2016), with 1,000 bootstrap replicates (▲ indicates the reference virus).

The full-length PRRSV genome and ORF5 gene have frequently been used for phylogenetic analysis (Shi et al., 2010b; Gao et al., 2017; Guo et al., 2018). PRRSV-2 can be divided into nine lineages (Lineages 1–9) based on ORF5 sequences, with inter-lineage genetic distances of 11–18% (Shi et al., 2010a,b; Gao et al., 2017). Lineage 1 is further divided into nine sublineages (Lineages 1.1–1.9) (Shi et al., 2010b). To explore the relationship between NADC34-like strains isolated in China and reference strains from other countries, a neighbor-joining phylogenetic tree was constructed based on whole-genome and ORF5-gene sequences. Both the whole-genome and ORF5-gene phylogenies showed that the NADC34-like strains isolated in China clustered within Lineage 1.5, forming a unique branch reflecting an introduction into China from the United States (Figure 2). This lineage differed from that of the RFLP 1-4-4 Lineage 1C variant associated with the recent PRRSV outbreak in the United States. Some NADC34-like strains (JLTZJ2050-2107, HLJTZJ2165-2108, HLJTZJ2007-2106, HLJTZJ1988-2106, and HLJTZJ2090-2107) were assigned to Lineage 1.8 (NADC30-like) based on whole-genome sequences because these strains were recombinant viruses whose parental strains fell into the Lineage 1.8 clade (Figure 2A and Supplementary Figure 1). However, these strains were classified into Lineage 1.5 in the ORF5-gene phylogeny. In addition, some strains (HLJZD22-1812, CH/SCYB-2/2020, and LNWK96) were assigned to Lineage 1.8 in the ORF5-gene analysis due to the insertion of genomic sequence from a Lineage 1.8 virus at the ORF5 gene position (Figure 2B). Thus, the use of the ORF5 gene to construct a phylogenetic tree has certain limitations.

The Nsp2 protein of PRRSV is the non-structural protein most prone to deletion and insertion mutations (Nelsen et al., 1999; Fang et al., 2004). Thus, deletions, insertions, and mutations in the Nsp2 region may represent ideal molecular markers for PRRSV. According to the latest research results, NSP2 has four distinct domains, namely N-terminal cysteine protease domain (PL2), central hypervariable domain (HV), transmembrane domain (TM), and C-terminal conserved region (Ziebuhr et al., 2000; Han et al., 2009). Previous studies have shown that Chinese HP-PRRSV exhibits a discontinuous 30 aa deletion at the Nsp2 position (1 aa + 29 aa) (Tian et al., 2007), NADC30-like PRRSVs are characterized by a discontinuous 131 aa deletion at the Nsp2 position (111 aa + 1 aa + 19 aa) (Brockmeier et al., 2012), and NADC34-like strains are characterized by a continuous deletion of 100 aa in the Nsp2 protein (Zhang et al., 2018). The deletion pattern of NSP2 is mainly concentrated in the HV domain (Li et al., 2021; Kim et al., 2022). Studies have shown that the virulence of HP-PRRSV is not directly related to discontinuous 30 amino acid deletions in the NSP2 gene (Zhou et al., 2009). But the PRRSV TJM vaccine strain (92th generations of TJ strain) showed another consecutive 120 amino acid deletion(NSP2 628aa–747aa)after the 30 amino acid deletion is related to its reduced virulence (Wang et al., 2013). And deletion of different regions of NSP2 can lead to significant changes in viral cell tropism (Wang et al., 2013). There are no studies to elucidate the relationship between the NSP2 deletion patterns and the virulence of NADC30-like PRRSV and NADC34-like PRRSV.

**FIGURE 3:**
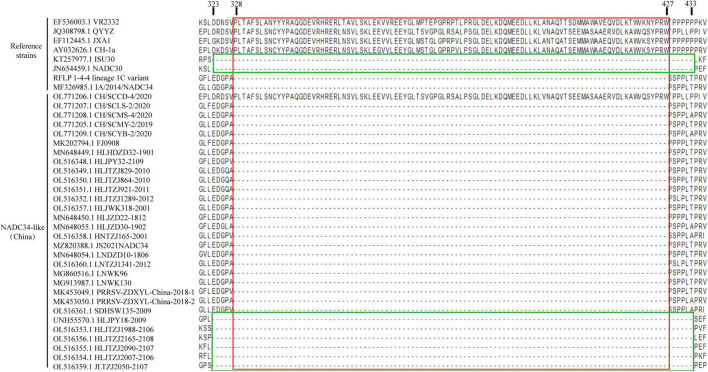
Alignment of Nsp2 amino acid sequences. Most of the NADC34-like strains isolated in China have a continuous deletion of 100 aa, which corresponds to positions 348–427 in Nsp2 from the reference strain VR2332 (boxed in red). The strains that recombine at the Nsp2 position differ (boxed in green). Amino acids were aligned using the MegAlign tool in Lasergene software (DNASTAR Inc., Madison, WI, United States).

In this study, the Nsp2 proteins of 30 NADC34-like isolates from China were analyzed using the MegAlign module of DNASTAR (Burland, 2000). A continuous 100-aa deletion was identified, corresponding to positions 328–427 of the VR2332 strain (Figure 3). In addition, some strains had foreign gene insertions at the Nsp2 position, resulting in a different deletion pattern from NADC34-like strains. Thus, this deletion pattern can serve as a molecular signature of NADC34-like strains and can be used for strain identification.

## Restriction fragment length polymorphisms demonstrated NADC34-like polymorphic complexity

**FIGURE 4:**
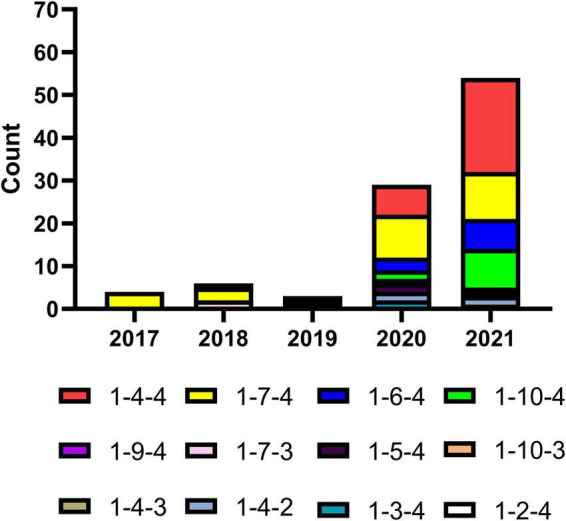
Restriction fragment length polymorphisms (RFLPs) in the ORF5 gene of NADC34-like strains in China. Some RFLPs in ORF5 had not previously been associated with *Hin*cII digestion. These patterns were artificially divided into patterns 9 and 10. The number of RFLP patterns identified in Chinese NADC34-like strains, as well as the relative abundances of patterns 1-4-4 and 1-7-4, increased between 2017 and 2021. The relative abundances of patterns 1-6-4 and 1-10-4, which were only reported recently, have also increased gradually. This graph was produced using Graphpad Prism V 7.03 (GraphPad Software Inc., San Diego, CA, United States).

Restriction fragment length polymorphisms (RFLPs) have been widely used in genome mapping, gene localization, taxonomy, and evolutionary analyses. RFLP analysis of ORF5 was used to differentiate a PRRSV vaccine strain from North American field strains (Wesley et al., 1998). All NADC34-like strains isolated in China were characterized and typed using the RFLP classification system previously described (Wesley et al., 1998). To obtain RFLPs, we performed three enzyme digestions (*Mlu*I, *Hin*cII, and *Sac*II) of all NADC34-like ORF5 genes and predicted them using Snapgene (Mante et al., 2021; Figure 4 and Supplementary Table 1). Across all RFLPs identified, two patterns had not been previously reported using *Hin*cII digestion (Wesley et al., 1998). These patterns were artificially divided into patterns 9 (*Hin*cII = nt 88, 219, 502) and 10 (*Hin*cII = nt 88, 219, 360, 502). Over time, the overall detection rate of NADC34-like strains increased significantly, as did the number of RFLP patterns. In particular, the relative abundance of RFLP patterns 1-4-4, 1-7-4, 1-6-4, and 1-10-4 increased rapidly to become the most abundant patterns in 2020–2021 (Figure 4). These results reflected the high genetic complexity of the NADC34-like strains and suggested that disease prevention and control will become more difficult over time. Notably, our results indicated that NADC34-like strains have exhibited explosive epidemic growth in China over the past 2 years (Figure 4).

## Recombination of NADC34-like strains increases viral complexity

Recombination is currently considered an important factor in the maintenance of PRRSV genetic diversity (Chen et al., 2013; Kappes and Faaberg, 2015). Because PRRSV lacks polymerase proofreading, viral replication is prone to errors and mutations, which may generate new strains that differ from the original with respect to virulence, pathogenesis, infectivity, and transmissibility (Shi et al., 2013; Brar et al., 2015; Zhao et al., 2015; Liu et al., 2017). The clinical implications of PRRSV recombination, as well as the overall minimization of viral recombination, are challenges that require solutions from the scientific community, followed by implementation by veterinary professionals (Risser et al., 2021). Here, we summarized the whole-genome NADC34-like data available in GenBank to date (i.e., submitted between 2017 and 2021). We then checked the associated literature to determine whether the strain was recombined and to note the clinical symptoms after recombination. Finally, we identified the parental strains, minor strains, and the position of recombination to clarify trends in the development of NADC34-like virulence (Table 1).

**TABLE 1:** Recombination information for NADC 34-like strains isolated in China.

No.	Strain	Accession no.	Whole genome (nt) (polyA tails removed)	Major parent	Minor parent	Reorganization area	Clinical signs	Isolation year	References
1	LNWK96	MG860516	15109	IA/2014/NADC34	ISU30 NADC30	1–1480 13193–15109	Abortion rate of 20% and piglet mortality rate of 10%	2017	Zhang et al., 2018
2	LNWK130	MG913987	15112	IA/2014/NADC34	ISU30	1–1480	Abortion rate of 30% and piglet mortality rate of 10%	2017	
3	FJ0908	MK202794	15112	IA/2014/NADC34	ISU30	760–1300	Abortion rate of 25% and piglet mortality rate of 40%	2018	Liu et al., 2019
4	HLJZD22-1812	MN648450	15109	IA/2014/NADC34	NADC30	13921–15109	Abortion rate of 20%	2018	Xu et al., 2020
5	LNDZD10-1806	MN648054	15112	IA/2014/NADC34	ISU30	1–1480	Abortion rate of 10%	2018	
6	CH/SCCD-4/2020	OL771206	15503	IA/2014/NADC34	QYYZ	1603–4052	Abortion rate of 10%	2020	Zhao et al., 2022
7	HLJPY18-2009	OL516347	15021	IA/2014/NADC34	NADC30	1–692	None reported	2020	Xu et al., 2022
					HuN4	693–1234			
					NADC30	1235–4205			
					NADC30	7817–9692			
8	HLJTZJ2007-2106	OL516354	15017	NADC30	HuN4	1–1504	None reported	2021	
					HuN4	4877–8561			
					IA/2014/NADC34	11587–15017			
9	HLJTZJ2165-2108	OL516356	15008	15HEN1	SDA3	1–698	None reported	2021	
					HLJZD30-1902	11709–14709			
10	HLJTZJ1988-2106	OL516353	15019	GXNN202004	LNDZD10-1806	13792–14168	None reported	2021	
11	JLTZJ2050-2107	OL516359	15019	HLJWK108-1711	IA/2014/NADC34	1266–1544	None reported	2021	
						5657–6331			
						11691–14261			
12	HLJTZJ2090-2107	OL516355	15009	QHD2	HLJWK108-1711	1–6409	None reported	2021	
					HLJWK108-1711	10403–11327			
					IA/2014/NADC34	13304–13785			
13	TJnh2021	Not public	15110	IA/2014/NADC34	QYYZ-like	12197–13627	Piglet morbidity 75% and mortality 40%	2021	Sun et al., 2022

Between 2017 and 2018, NADC34-like strains primarily recombined with strains from the United States, not native Chinese strains. However, NADC34-like strains recombined with a Chinese native strain after the year 2020. These recombinant strains clustered with Lineage 1.8 (15HEN1/GXNN202004/QHD2/HLJWK108-1711), Lineage 3.5 (QYYZ), and Lineage 8.7 (HuN4/SDA3). The frequency of recombination within lineages was higher than that among lineages. Notably, recombination was most frequent between Lineage 1.5 (NADC34-like) and Lineage 1.8 (NADC30-like) (Table 1). NADC34-like strains have an extremely complex recombination pattern, with most of the recombination breakpoints located in Nsp1 (4), Nsp2 (7), Nsp3 (1), Nsp5 (1), Nsp7 (1), Nsp9 (1), Nsp11 (1), Nsp12 (1), ORF2 (3), ORF4 (2), and ORF5 (2).

**FIGURE 5:**
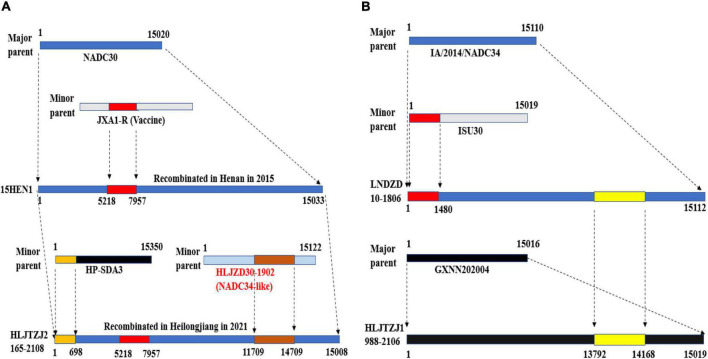
Schematic diagram of the recombination of HLJTZJ2165-2108, LNDZD10-1806, and HLJTZJ1988-2106 strains. **(A)** Origin of HLJTZJ2165-2108 strain. **(B)** Origin of strains LNDZD10-1806 and HLJTZJ1988-2106. Maps produced using Adobe Illustrator CC 2019 (Adobe Inc., San Jose, CA, United States).

The HLJTZJ2165-2108 strain and the HLJTZJ1988-2106 strain have complex recombination structures, suggesting that chimeric strains can continue to recombine after encountering other strains (Figure 5). The 15HEN1 strain was obtained through the recombination of the NADC30 strain (major parent) and the JXA1-R vaccine virus (minor parent) in Henan in 2015. This strain spread from Henan to Heilongjiang and there recombined with the HP-ADA3 strain (minor parent) and the HLJZD30-1902 strain (minor parent) to form the HLJTZJ2165-2108 strain (Figure 5A) (Zhao et al., 2017; Xu et al., 2022). The LNDZD10-1806 strain, which was formed through the recombination of the IA/2014/NADC34 strain (major parent) and the ISU30 strain (minor parent), spread to Heilongjiang and recombined with the GXNN202004 strain (major parent) to generate the HLJTZJ1988-2106 strain (Figure 5B) (Xu et al., 2020, 2022). It is unclear whether the LNDZD10-1806 strain recombined domestically or was introduced into China after recombination in the United States. The complex recombination pattern of NADC34-like PRRSV is similar to that of NADC30-like strains (Zhang et al., 2019).

Recombination leads to new strains with varying pathogenicity. For example, recent findings suggest that the recombinant strain TJnh2021 has higher morbidity and lethality than previously discovered NADC34-like strains (Table 1). These results demonstrated that NADC34-like PRRSV recombines with strains of different subtypes, resulting in inconsistent virulence among the recombinant strains and thus posing a challenging obstacle to the effective prevention and treatment of PRRSV (Chen et al., 2018, 2021).

## Pathogenicity of NADC34-like strains is variable

The NADC34-like (RFLP 1-7-4) lineage, which emerged in the United States in 2014, is a highly pathogenic strain that causes dramatic abortion ‘storms’ in sow herds and high mortality among piglets (Alkhamis et al., 2016; van Geelen et al., 2018). Studies have shown that the NADC34-like PRRSV strains IA/2014/NADC34, IA/2013/ISU-1, and IN/2014/ISU-5 can cause more severe disease (van Geelen et al., 2018). In contrast, strain IA/2014/ISU-2 has deleterious effects and does not even cause typical PRRSV symptoms such as fever (van Geelen et al., 2018). This indicates that there are significant differences in the pathogenicity of NADC34-like PRRSV in the United States (van Geelen et al., 2018).

Similarly, Xie C.Z. et al. (2020) showed that the pathogenicity of the Chinese PRRSV isolate PRRSV-ZDXYL-China-2018-1 was low, with infected piglets exhibiting mild clinical symptoms at 2–6 days post-challenge (dpc), including depression, cough, and anorexia, while piglets inoculated with isolate HP-LQ-JXA1-like exhibited more severe clinical symptoms at 2–14 dpc, such as high fever and dyspnea (Xie C.Z. et al., 2020). At 14 dpc, one-fifth of the piglets inoculated with the HP-LQ-JXA1-like strain had died (mortality 20%), while no deaths occurred in the same time frame among the control piglets and those inoculated with strain PRRSV-ZDXYL-China-2018-1. Pathological observations indicated that the piglets inoculated with HP-LQ-JXA1-like strain were characterized by severe interstitial pneumonia and secondary infections, while the PRRSV-ZDXYL-China-2018-1 group exhibited moderate to severe interstitial pneumonia with microscopic lesions, characterized by type II pneumocyte proliferation and accumulation of cellular debris in the alveoli. Immunohistochemistry (IHC) identified positive brown-red epithelial cells and macrophages in the lungs of the piglets infected with PRRSV-ZDXYL-China-2018-1 and LQ-JXA1-like strains, but not in the lung tissues of the piglets in the control group. According to the mortality rate, it demonstrated that PRRSV-ZDXYL-China-2018-1 strain was only moderately pathogenic and was less severe than the HP-LQ-JXA1-like strain (Xie C.Z. et al., 2020).

Independently, Song et al. (2020) evaluated the pathogenicity of HLJDZD32-1901 strain and showed that infected piglets had no fever and death (mortality 0%), exhibiting only coughing and anorexia compared with the control group. The body weights of the challenge group and the control group remained similar at 0–7 dpc. However, daily gain in the challenge group was lower than that in the control group at 8–14 dpc. In the challenge group, serum antibodies were partially positive at 7 dpc and completely positive at 10 dpc, while the antibodies of the control group remained negative. Viraemia was detected in some piglets in the challenge group at 3 dpc, peaking at 7 dpc. Viral loads were highest in the lungs, followed by the lymph nodes and the tonsils. In the lung tissues of the challenge group, pathological observation identified obvious hemorrhagic spots and parenchymal lesions, while immunohistochemistry revealed brown-red macrophages. The lung tissues of the control group were normal. According to the mortality rate, it indicates that HLJDZD32-1901 was mildly pathogenic to piglets. However, based on clinical morbidity, it was speculated that HLJDZD32-1901 might be highly pathogenic to pregnant sows (Song et al., 2020).

Our analysis showed that PRRSV-ZDXYL-China-2018-1 and HLJDZD321901 were moderate virulence and low virulence, respectively, and that neither strain was recombinant. However, NADC34-like strains recombine with other strains, resulting in unpredictable changes in virulence. For example, in 2021, a strain with high pathogenicity to piglets was isolated in Tianjin (named TJnh2021) (Sun et al., 2022). TJnh2021 was determined to be a natural recombinant of IA/2014/NADC34 (major parent) and QYYZ (minor parent) (Table 1). Pathogenicity tests showed that piglets inoculated with TJnh2021 developed fever and viremia, as well as other obvious clinical signs, including cough, anorexia, and reddish-purple body and ears, at 3 dpc. Two piglets (of the five challenged) died within 12 dpc (mortality 40%). In the infected piglets, lung lesions exhibited pulmonary consolidation, and histopathology revealed interstitial pneumonia; the lungs of the control group were normal (Sun et al., 2022). According to the mortality rate, it indicated that the pathogenicity of the recombinant strain TJnh2021 was greater than the pathogenicity of the non-recombinant strains PRRSV-ZDXYL-China-2018-1 and HLJDZD32-1901 (Song et al., 2020; Xie C.Z. et al., 2020), demonstrating that there are also significant differences in pathogenicity among the NADC34-like strains isolated in China. Currently, the pathogenicity of NADC34-like strains in pregnant sows is unknown. Only by fully understanding the pathogenicity of these viral strains in both piglets and pregnant sows, we can better understand and control PRRSV.

## Porcine reproductive and respiratory syndrome virus vaccine status in China

Vaccination is the most effective and practical way to prevent and control infectious diseases (Canoui and Launay, 2019). Currently, six commercial PRRSV vaccines are widely used in China: CH-1R, JXA1P80, HuN4-F112, GDr180, TJM-F92, and Resp PRRS MLV (all named based on the corresponding strain) (Bai et al., 2016; Wang et al., 2016). However, the repeated outbreaks of PRRS and the emergence of new PRRSV variants indicate that current vaccines are not fully effective. Indeed, the commercial PRRSV live vaccines currently available are protective against homologous strains but confer limited protection against heterologous strains (Charerntantanakul, 2012; Roca et al., 2012; Kick et al., 2019). In contrast to MLV vaccines, inactivated PRRSV vaccines are safe and have thus been licensed worldwide. However, several studies have demonstrated that inactivated PRRSV vaccines are ineffective against wild-type infections as they neither trigger the production of specific PRRSV antibodies nor cell-mediated immunity (CMI) responses (Bassaganya-Riera et al., 2004; Kim et al., 2011; Yu et al., 2019; Wang et al., 2021). Li et al. evaluated the cross-protective efficacy of the synergy between live-attenuated and inactivated PRRSV vaccines compared with a single vaccination with PRRS modified-live virus (MLV) vaccine against NADC30-like strain. Results indicated that MLV provides substantial cross-protection against the NADC30-like virus, and the inactivated vaccine confers to additional immune effect when live-attenuated combined with inactivated PRRSV vaccines in piglets as a booster (Li et al., 2022). Another study showed that repeated immunization with PRRSV inactivated vaccine in sows with high PRRSV seroprevalence (naturally infected PRRSV) can not only significantly improve their reproductive performance but increase the survival rate of weaned piglets and the secretion of IFN-γ. It has proved that the synergistic effect of the live vaccine and the inactivated vaccine can promote a cellular immune response (Renukaradhya et al., 2015). Indeed, commercially available live vaccines do not provide good protection against PRRSV strains in Lineage 1, including NADC30-like PRRSV (Sublineage 1.8) and NADC34-like PRRSV (Sublineage 1.5) (Bai et al., 2016; Zhou et al., 2021). The vaccines available in China are primarily effective against strains in Lineage 5 or Lineage 8. However, in recent years, the epidemic lineage of PRRSV-2 strain in China has shifted from Sublineage 8.7 (CH-1a-like and HP-PRRSV-like) to Sublineage 1.8 (NADC30-like) and Sublineage 1.5 (NADC34-like) (Li et al., 2021; Xu et al., 2022). The large genetic distances between vaccine lineages and the lineages of the epidemic strains may be one reason why existing vaccines fail to protect against epidemic strains. Recently, a NADC30-Like PRRSV vaccine candidate targeting lineage 1 has been developed. With the continuous spread of NADC34-like in China, there is an urgent need to develop candidate vaccines against NADC34-like PRRSV (Zhang et al., 2022). In addition, many known issues with PRRS vaccines remain unresolved, such as cross-protection and safety. Therefore, there is an urgent need to develop a genetically stable chimeric vaccine targeting the existing epidemic strain lineages and conferring resistance to various mutant strains to control PRRS more effectively (Ellingson et al., 2010; Sun et al., 2016; Tian et al., 2017; Su et al., 2019).

## Conclusion and perspectives

(1) NADC34-like strain has become the major epidemic strain in some Chinese provinces and is spreading nationwide.
(2) The main epidemic strains vary among Chinese provinces. Abortion rates, mortality, and recombination breakpoints vary among NADC34-like strains as well as their recombinants.
(3) The current intensive aquaculture pattern in China leads to high stocking density, so local strains are popular in the populations faster. The continuous introduction of foreign breeding swine leads to the recombination of new strains with local epidemic strains, which promotes the diversity of PRRSV. To control PRRS more effectively, the introduced breeding swine should be isolated and detected in time, and new strains found should be treated harmlessly to clear the populations and purify PRRS.
(4) In particular, pig farms should continuously monitor the prevalence of NADC34-like PRRSV (PRRSV 1-4-4 lineage 1c variant) to prevent the emergence of new virulent mutants.

## Author contributions

All authors listed have made a substantial, direct, and intellectual contribution to the work, and approved it for publication.

## Conflict of interest

The authors declare that the research was conducted in the absence of any commercial or financial relationships that could be construed as a potential conflict of interest.

## Publisher’s note

All claims expressed in this article are solely those of the authors and do not necessarily represent those of their affiliated organizations, or those of the publisher, the editors and the reviewers. Any product that may be evaluated in this article, or claim that may be made by its manufacturer, is not guaranteed or endorsed by the publisher.
