# Supplementary figures and images for: Recent advances in the study of NADC34-like porcine reproductive and respiratory syndrome virus in China
Source: Front Microbiol. 2022 Jul 22;13:950402. doi: 10.3389/fmicb.2022.950402 (PMC9354828; doi:10.3389/fmicb.2022.950402)

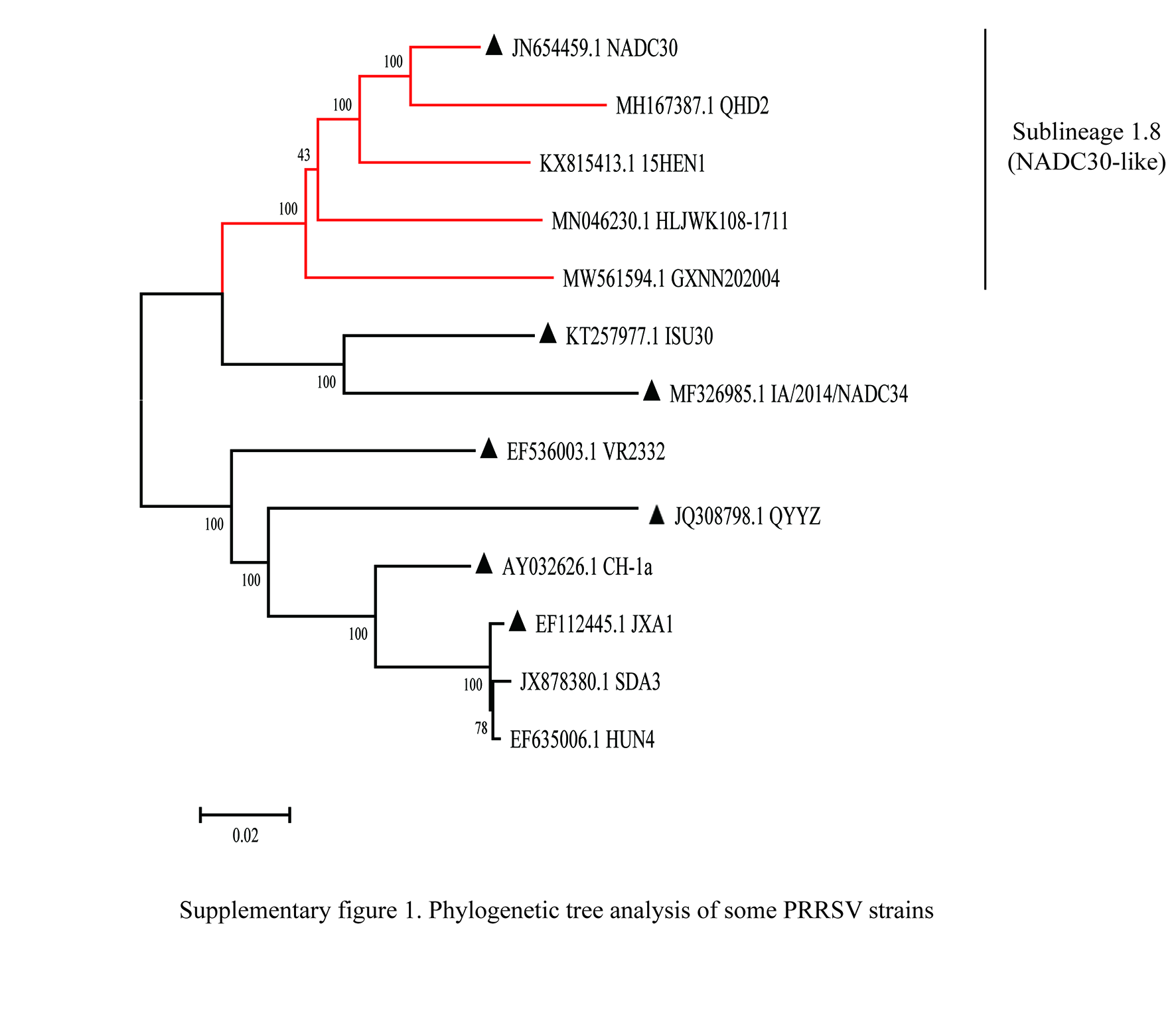

Supplement: Supplementary file 2 [file Image_1.tif]
